# 2023 Korean Society of Echocardiography position paper for the diagnosis and management of valvular heart disease, part II: mitral and tricuspid valve disease

**DOI:** 10.1186/s44348-024-00021-6

**Published:** 2024-06-25

**Authors:** Chi Young Shim, Eun Kyoung Kim, Dong-Hyuk Cho, Jun-Bean Park, Jeong-Sook Seo, Jung-Woo Son, In-Cheol Kim, Sang-Hyun Lee, Ran Heo, Hyun-Jung Lee, Sahmin Lee, Byung Joo Sun, Se-Jung Yoon, Sun Hwa Lee, Hyung Yoon Kim, Hyue Mee Kim, Jae-Hyeong Park, Geu-Ru Hong, Hae Ok Jung, Yong-Jin Kim, Kye Hun Kim, Duk-Hyun Kang, Jong-Won Ha, Hyungseop Kim, The Korean Valve Survey Investigators, Wook-Jin Chung, Chan Seok Park, Hyo-Suk Ahn, Woo-Baek Chung, Eun Joo Cho, Jung Sun Cho, Dong Ryeol Ryu, Dong Heon Yang, Jeong Rang Park, Woo-Shik Kim, Il Suk Sohn, Jin Oh Na, Seong-Mi Park, Sun Ho Hwang, Ji-Yong Choi, Tae-Ho Park, Yong Hyun Park, Jung Hyun Choi, Hack-Lyoung Kim, Hye Sun Seo, Jin-Sun Park, Eui-Young Choi, Jang-Won Son, Shin-Jae Kim, Sang Jae Rhee, In-Jeong Cho, Young Sup Byun, Sung-Hee Shin, Sun Hwa Lee, Jong Wook Beom, Ju-Hee Lee, Dae-Hwan Bae, Sung-Ai Kim, Dae Gyun Park, Min-Kyung Kang, Kyung-Soon Hong

**Affiliations:** 1 Division of Cardiology, Severance Cardiovascular Hospital, Yonsei University College of Medicine, Seoul, Republic of Korea; 2 Division of Cardiology, Department of Internal Medicine, Heart Vascular Stroke Institute, Samsung Medical Center, Sungkyunkwan University School of Medicine, Seoul, Republic of Korea; 3 Division of Cardiology, Department of Internal Medicine, Korea University Anam Hospital, Seoul, Republic of Korea; 4 Division of Cardiology, Department of Internal Medicine, Seoul National University Hospital, Seoul National University College of Medicine, Seoul, Republic of Korea; 5 Division of Cardiology, Department of Internal Medicine, Inje University Busan Paik Hospital, Inje University College of Medicine, Busan, Republic of Korea; 6 Division of Cardiology, Department of Internal Medicine, Yonsei University Wonju College of Medicine, Wonju, Republic of Korea; 7 Division of Cardiology, Department of Internal Medicine, Keimyung University Dongsan Medical Center, Daegu, Republic of Korea; 8 Division of Cardiology, Department of Internal Medicine and Research Institute for Convergence of Biomedical Science and Technology, Pusan National University Yangsan Hospital, Pusan National University School of Medicine, Yangsan, Republic of Korea; 9 Division of Cardiology, Department of Internal Medicine, Hanyang University College of Medicine, Seoul, Republic of Korea; 10 Division of Cardiology, Asan Medical Center, University of Ulsan College of Medicine, Seoul, Republic of Korea; 11 Division of Cardiology, National Health Insurance Service Ilsan Hospital, Goyang, Republic of Korea; 12 Department of Cardiology, Jeonbuk National University Hospital, Jeonbuk National University Medical School, Jeonju, Republic of Korea; 13 Department of Cardiovascular Medicine, Chonnam National University Hospital, Gwangju, Republic of Korea; 14 Division of Cardiology, Department of Internal Medicine, Chung-Ang University Hospital, Chung-Ang University College of Medicine, Seoul, Republic of Korea; 15 Division of Cardiology, Department of Internal Medicine, Chungnam National University Hospital, Chungnam National University College of Medicine, Daejeon, Republic of Korea; 16 Division of Cardiology, Department of Internal Medicine, Seoul St. Mary’s Hospital, College of Medicine, The Catholic University of Korea, Seoul, Republic of Korea

**Keywords:** Mitral stenosis, Mitral regurgitation, Tricuspid regurgitation

## Abstract

This manuscript represents the official position of the Korean Society of Echocardiography on valvular heart diseases. This position paper focuses on the diagnosis and management of valvular heart diseases with referring to the guidelines recently published by the American College of Cardiology/American Heart Association and the European Society of Cardiology. The committee sought to reflect national data on the topic of valvular heart diseases published to date through a systematic literature search based on validity and relevance. In the part II of this article, we intend to present recommendations for diagnosis and treatment of mitral valve disease and tricuspid valve disease.

## Background

This manuscript represents the official position of the Korean Society of Echocardiography (KSE) on valvular heart diseases. This position paper focuses on the diagnosis and management of valvular heart diseases with reference to the guidelines recently published by the American College of Cardiology/American Heart Association (ACC/AHA) [1] and the European Society of Cardiology/European Association for Cardio-Thoracic Surgery (ESC/EACTS) [2]. The committee sought to reflect national data on the topic of valvular heart diseases published to date through a systematic literature search based on validity and relevance. In part II of this article, we intend to present recommendations for diagnosis and treatment of mitral valve (MV) disease and tricuspid valve disease.

## Mitral stenosis

### Etiology

#### Rheumatic MS

The causes of mitral stenosis (MS) are mostly rheumatic or degenerative. Rheumatic fever is a poststreptococcal immune-mediated inflammatory disease, which results in a high degree of valve fibrosis and dysfunction through long-term inflammation, predominantly involving the MV, and sometimes causing multiple valve disease. According to the statistics of the global, regional, and national burden of rheumatic heart disease published in 2017 [3], the health-related burden of rheumatic heart disease has declined worldwide, but high rates of the disease persist in some underdeveloped regions in the world. In the 2015 statistics, Republic of Korea (hereinafter, Korea) had a very low prevalence [3]. Although there have been few new patients with rheumatic fever owing to the rapid improvement in socioeconomic status from the 1950s to the present, Korea still has a large number of aging patients with rheumatic MS because Korea is one of the developed countries that can systematically apply the latest imaging diagnosis and treatment for the disease [4]. Percutaneous mitral valvuloplasty (PMV) was started by Inoue et al. [5] in 1984 and has been established as an effective treatment for patients with symptomatic rheumatic MS for more than 30 years. Especially in Korea, some leading centers have accumulated extensive clinical experience on rheumatic MS and PMV [6–10].

#### Degenerative MS

There has been a gradual transition in the epidemiology of MS in the Western world, with rheumatic disease in rapid decline and an increasing recognition of degenerative mitral annular calcification (MAC)-related MS in the elderly [11]. A similar phenomenon is occurring in Korea as well [4]. MAC is a chronic degenerative process affecting the fibrous skeleton of the mitral ring [12, 13]. The main contributing factors for occurrence of MAC are age-related degeneration, elevated left ventricular afterload, atherosclerotic risk factors, and aberrant calcium-phosphate metabolism [14–18]. In the past, MAC was considered an incidental finding in the elderly population or in those with chronic kidney disease. However, growing evidence shows that MAC is associated with cardiovascular morbidity and mortality [19–22]. In addition, patients with hemodynamically significant MAC resulting in MS showed poor clinical outcomes and higher incidence of atrial fibrillation (AF) and stroke [15, 23]. Through several recent investigations [24, 25], the differences in clinical factors, echocardiographic characteristics and pathophysiology between rheumatic MS and MAC-related degenerative MS have been demonstrated as shown in Table 1.

**Table 1:** Characteristics of rheumatic MS versus MAC-related degenerative MS

Characteristic	Rheumatic MS	MAC-related degenerative MS
Anatomy	Funnel-shaped geometry	Tubular orifice geometry
	Commissural fusion	Commissures spared
Epidemiology	Younger population	Elderly, comorbid population
Echo assessment	MVA quantification validated	MVA quantification challenging
		Greater MVA relative to mean gradient than rheumatic MS
Treatment	PMV if favorable valve morphology without contraindication	Poor valvuloplasty candidates
	MV replacement	Transcatheter or surgical MV replacement

*MS* Mitral stenosis, *MAC* Mitral annular calcification, *MVA* Mitral valve area, *PMV* Percutaneous mitral valvuloplasty

### Stages

The stages of MS are defined by patient symptoms, valve anatomy, valve hemodynamics, and the consequences of valve obstruction on the left atrium (LA) and pulmonary circulation (Table 2).

**Table 2:** Stages of mitral stenosis

Characteristic	Stage				
	A	B		C	D
Definition	At risk	Progressive		Asymptomatic severe	Symptomatic severe
Severity	Normal to insignificant	Mild	Moderate	Severe	
Echocardiography morphology					
Leaflet	Mildly fibrotic or sclerotic, mild doming	Partial fibrothickening, sclerocalcified, doming		Severe fibrothickening, sclerocalcified, doming	
Commissure	No fusion	Mild to moderate fusion		Severe fusion	
Annulus	Mild calcification	Mild to moderate calcification		Severe calcification	
Area (cm^2^)	-	> 2.5	1.6–2.5	≤ 1.5 (very severe, ≤ 1.0)	
MDPG (mmHg)	-	< 3	3–5	≥ 5 (very severe, ≥ 10)	
PHT (msec)	-	< 100	100–149	≥ 150	
PASP (mmHg)	Normal	Normal	Normal at rest	Normal at rest or > 50	> 50
LA size	Normal	Normal or ↑	↑	↑	
Symptom	None	None		None	DOE, EI

*MDPG* Mean diastolic pressure gradient, *PHT* Pressure half-time, *PASP* Pulmonary artery systolic pressure, *LA* Left atrium, *DOE* Dyspnea on exertion, *EI* exercise intolerance

The definition of “severe” MS is based on the severity of symptoms, as well as the severity at which intervention will improve symptoms. Thus, an MV area (MVA) ≤ 1.5 cm^2^ is considered severe, which typically corresponds to a transmitral mean diastolic pressure gradient (MDPG) greater than 5 mmHg at a normal heart rate. However, MDPG is highly dependent on transvalvular flow rate, diastolic filling period, and heart rate. Mitral pressure half-time also has limitations and is dependent upon left ventricular (LV) and LA compliance as well as stenosis severity. It is true that confusion has occurred in diagnosis and treatment criteria while correcting the cutoff value of the definition of severe MS to MVA of 1.5 cm^2^ from 1.0 cm^2^ in the AHA/ACC guideline in 2014 [26] and its focused update in 2017 [27].

According to the practice survey conducted by the KSE in 2022, 62.1% of institutions are still using the MVA cutoff of 1.0 cm^2^ for severe MS and 37.9% of institutions are complying with the cutoff of 1.5 cm^2^ [1, 2]. The reasons for sticking to the original criteria were diverse: lack of sufficient evidence for changes in cutoff values, worsening discrepancy between MVA and MDPG, the view that MVA of 1.5 cm^2^ as the tipping point for intervention was unreasonable, and inevitable confusion in patients on regular follow-up whose MS severity would be newly classified due to changes in cutoffs rather than progression of valve disease.

The KSE guideline committee has developed expert consensus on this issue through structured face-to-face meetings. In this position paper, MVA of 1.5 cm^2^ is determined as the cutoff for severe MS in rheumatic MS. In addition, the criterion for very severe MS is determined to be MVA ≤ 1.0 cm^2^. In cases of degenerative MS, that is, MAC-related MS, the role of MDPG is more emphasized, but the severity diagnostic criteria are equally recommended. As a diagnostic criterion for severe MS, the MVA standard refers to structural abnormalities that suggest the need for intervention. However, decision-making should be made based on the patient’s symptoms and hemodynamic consequences, such as pulmonary hypertension or atrial arrhythmia.

The cutoff values for MDPG correlating with MS severity were set to reduce the discrepancy between MVA and MDPG. When there is a discrepancy between MVA and MDPG, it is recommended to apply the biplane method or three-dimensional (3D) multiplanar reconstruction to obtain accurate measurement of MVA. In cases of degenerative MS due to MAC or functional MS due to other causes, it may be difficult to measure the MVA, so the clinical significance may have to be interpreted according to hemodynamic parameters.

### Korean data

A recent nationwide hospital-based retrospective cohort study from the Korean Valve Survey collected clinical and echocardiographic data on valvular heart disease from 45 medical centers [28]. Among 4,089 patients, 244 patients showed MS and mean age of MS patients was 65.5 ± 10.9 years, which was significantly younger than those with mitral regurgitation (MR) or other valvular dysfunction. 74.6% of patients with MS had rheumatic etiology and 20.5% had degenerative causes.

Historically, abundant research on rheumatic MS has been reported in Korea [6–10, 29–32]. Kang et al. [29] compared the long-term outcomes of early preemptive PMV and a conventional treatment strategy to define the optimal timing of PMV in asymptomatic patients with moderate MS. At the time of conducting this study, the guidelines defined MVA 1.0 to 1.5 cm^2^ as moderate MS, so if interpreted using the current guidelines, this study was in fact about asymptomatic severe MS. In 244 asymptomatic patients with severe rheumatic MS (MVA, 1.0–1.5 cm^2^) who were potential candidates for early PMV, the risk of cardiovascular endpoint was significantly lower in the PMV group than in the conventional group after propensity matching. They concluded that the clinical benefits of early PMV may outweigh the risks associated with early intervention, but prospective randomized trials are required to confirm the efficacy of early PMV. Looking at the 20-year experience of a single-center study reported by Kim et al. [8] in 2018, the factors affecting the long-term outcome of PMV were echo score > 8 and post-PMV MVA of 1.76 cm^2^ or less. A recent report on 10-year trends in the incidence, treatment, and outcomes based on the Korean Health Insurance Review and Assessment Service (HIRA) database showed that the overall incidence of MS in Korea has decreased remarkably [30]. The data also demonstrated the increasing rate of anticoagulant use and similar incidence of systemic embolism, but increasing incidence of intracranial hemorrhage, suggesting a topic that should be considered in the treatment of MS in the future. To overcome the limitations of the previous retrospective study on early PMV in asymptomatic MS, a prospective randomized multicenter trial was conducted in Korea [31]. However, the effect of early PMV was not demonstrated in asymptomatic MS patients. In addition, a study on the trajectory profile of rheumatic MS was conducted in Korea to find factors that predict rapid progression [32]. In a total of 436 patients with severe MS with a valve area of 1.0 to 1.5 cm^2^, rapid progression of pulmonary artery systolic pressure determined prognosis, which was associated with a pulmonary artery systolic pressure of 40 mmHg on initial evaluation.

Recently, with an increase in the elderly population in Korea, research on degenerative MS and MAC is being actively conducted [15, 17, 21]. In particular, a study on the sex differences of risk factors in patients with MAC has been reported [21]. In hemodynamic comparisons between degenerative MS and rheumatic MS, degenerative MS presented with a greater MVA relative to MDPG than rheumatic MS [25]. Kim et al. [15] reported the clinical significance of morphological and structural characteristics of MAC. Morphological and functional features of MAC on echocardiogram were scored from 0 to 3 according to MAC mobility, presence of echo-dense mass with central echolucencies in the periannular region suggesting caseous necrosis, and functional stenosis in 460 subjects with MAC. They demonstrated that this MAC score was independently associated with stroke and had significant incremental value over demographic factors and comorbidities for predicting stroke. In the future, studies that more objectively assess MAC severity using cardiac computed tomography (CT) are expected to be conducted.

### Diagnosis and follow-up

#### Rheumatic MS

1. Diagnosis

For patients with rheumatic MS, there is no doubt that transthoracic echocardiography (TTE) is a fundamentally important first-line imaging for evaluating structure and function in MS. In particular, it plays an important role in evaluating the severity of MS, determining feasibility of intervention, and classifying risk after intervention. The hallmark feature of rheumatic MS is commissural fusion, leading to the classic “fish mouth” appearance of the MV orifice, and thickening and restriction of the posterior leaflet. Notably, calcification in rheumatic MS primarily involves the leaflet tip, which is distinguishable from annular calcification found in degenerative MS. Among many echocardiographic parameters, MVA using 2D planimetry is the reference method of measuring MS severity, and severe MS is defined by an MVA ≤ 1.5 cm^2^ [1, 2]. Planimetry by 3D echocardiography may have an additional diagnostic value by permitting accurate identification of the MV orifice. Transmitral MDPG and pulmonary arterial pressures are useful parameters reflecting hemodynamic consequences of MS and providing prognostic information [33].

Table 3 shows the advantages, disadvantages, and indications of additional diagnostic tests in patients with MS. Stress testing is recommended in patients with no or equivocal symptoms or discordant symptoms with MS severity measured at rest. Exercise echocardiography has a role in these patients for the evaluation of changes in transmitral MDPG and pulmonary artery pressure. Dobutamine stress echocardiography is also a safe and feasible stress test; however, it has some limitations compared to exercise echocardiography [34]. The role of dobutamine stress echocardiography in these patients is primarily to assess contractile reserve and myocardial ischemia, which could contribute to symptoms or help identify underlying coronary artery disease. It can also provide information on the hemodynamic response to stress in patients who are unable to exercise or have limited exercise capacity. Nonetheless, exercise echocardiography is often considered superior to dobutamine stress echocardiography in the evaluation of MS, as dobutamine induces changes in loading conditions that are different from exercise-induced changes and thus does not simulate day-to-day physiologic stress as accurately. Cardiac catheterization is useful in patients with discordant grading of MS to further characterize hemodynamics and the cause of symptoms, as this test allows measurement of absolute pressures in the LA, LV, and pulmonary circulation at rest and even during exercise. Although TTE usually provides sufficient information for routine management of patients with rheumatic MS, transesophageal echocardiography (TEE) is superior to TTE in obtaining detailed information on mitral anatomy, particularly in assessing commissural zones, subvalvular apparatus, and other complex MV pathology. For this reason, TEE should be performed to determine suitability for PMV [35, 36], and also to exclude the presence of thrombus in LA or LA appendage before PMV.

**Table 3:** Comparison of diagnostic tools for the evaluation of MS

Test	Advantage	Disadvantage	Indication
Exercise SE	Physiological stress test	Limited in patients unable to exercise	Evaluation of exercise hemodynamics associated with MS
	Minimal invasiveness		Objective assessment of symptoms and exercise capacity
Dobutamine SE	Relative ease of performance compared to exercise SE	Nonphysiological stress test	Assessment of contractile reserve and myocardial ischemia/viability
	Minimal invasiveness	Risk of dobutamine-induced arrhythmias	
Cardiac catheterization	Absolute pressure measurements	Risk of invasive complications: bleeding, infection, vascular damage	Possible confirmatory test for discordant or inconclusive results of MS severity grading
	Hemodynamic assessment during exercise		Evaluation of hemodynamics responsible for exertional symptoms
TEE	Provision of high-resolution images	Semi-invasiveness	More thorough evaluation of valve morphology and function
	Better visualization of mitral valve, LA, and LAA	Patient discomfort	Detection of thrombus in LA or LAA

*MS* Mitral stenosis, *SE* Stress echocardiography, *TEE* Transesophageal echocardiography, *LA* Left atrium, *LAA* Left atrial appendage

(2) Follow-up

As rheumatic MS is a slowly progressive disease, repeated TTE at intervals dictated by valve area is recommended for patients with asymptomatic MS. In patients with mild MS, although the rate of further narrowing is quite variable [37], the average rate of progression is a decrease in valve area of 0.1 cm^2^/yr [38]. However, it is important to note that progressive increases in right ventricular (RV) size and RV systolic pressure can be found, even in the absence of a decrease in MVA. Intervals of 2 to 3 years are appropriate in cases of moderate MS [1]. Close surveillance of disease progression with yearly TTE is recommended in asymptomatic patients with severe MS. Follow-up strategies for patients undergoing successful PMV are similar to those of asymptomatic patients and more frequent follow-up visits and evaluations are indicated if restenosis occurs even though patients remain asymptomatic.

#### Degenerative MS

1. Diagnosis

Defining the severity of degenerative MS is difficult and the lack of validation of the usual parameters used in rheumatic MS is still an issue. For instance, extensive calcification and irregular orifice of the MV hamper the accurate measurement of MVA, making it less reliable [39, 40]. Furthermore, abnormal LA and LV compliance, which are commonly present in patients with degenerative MS, cause a high transmitral MDPG even in the absence of significant MS [11]. Although MDPG is not useful for determining disease severity and threshold for intervention, this echocardiographic parameter may have prognostic value in patients with degenerative MS [23]. Echocardiography remains the initial imaging modality of choice for the assessment of MS when an intervention is planned, and cardiac CT is often necessary to evaluate the location and degree of calcification and to determine the feasibility of a planned intervention [41].

(2) Follow-up

Data is scarce on the rate of progression in degenerative MS and the factors influencing progression rate. Evidence is lacking to support a recommendation for follow-up intervals in patients with degenerative MS, but follow-up may be performed similarly to rheumatic MS.

### Medical therapy

Diuretics can improve symptoms by reducing LA pressure and pulmonary congestion. β-blockers, nondihydropyridine calcium channel blockers, and digoxin can also improve symptoms by prolonging diastole and consequently improving LV filling at rest and during exercise.

In patients with AF, anticoagulation with a vitamin K antagonist with a target international normalized ratio of 2 to 3 is recommended. Importantly, vitamin K antagonists should be preferred over direct oral anticoagulants in patients with moderate-to-severe rheumatic MS and AF. A recent study using the HIRA database suggests that the use of direct oral anticoagulants is promising in the prevention of thromboembolic events in patients with MS and AF [42], but there is no solid evidence to support this yet. In patients in sinus rhythm, an oral anticoagulant is recommended in the setting of prior systemic embolism or presence of a thrombus in the LA [43]. However, it is controversial whether long-term oral anticoagulation should be considered when there is only LA enlargement or spontaneous echocardiographic contrast on TEE [44, 45]. The benefit of rhythm control for AF should be considered based on severity of MS, LA size, and possibility of sinus conversion. In patients with significant MS, rhythm control strategies including cardioversion and ablation are not recommended before relieving MS because of the low probability of achieving durable restoration of sinus rhythm. However, cardioversion should be performed immediately after successful intervention in patients with AF of recent onset and moderately enlarged LA. Among antiarrhythmic medications, amiodarone is most effective in maintaining sinus rhythm after cardioversion of AF.

### Timing of intervention

**Fig. 1:**
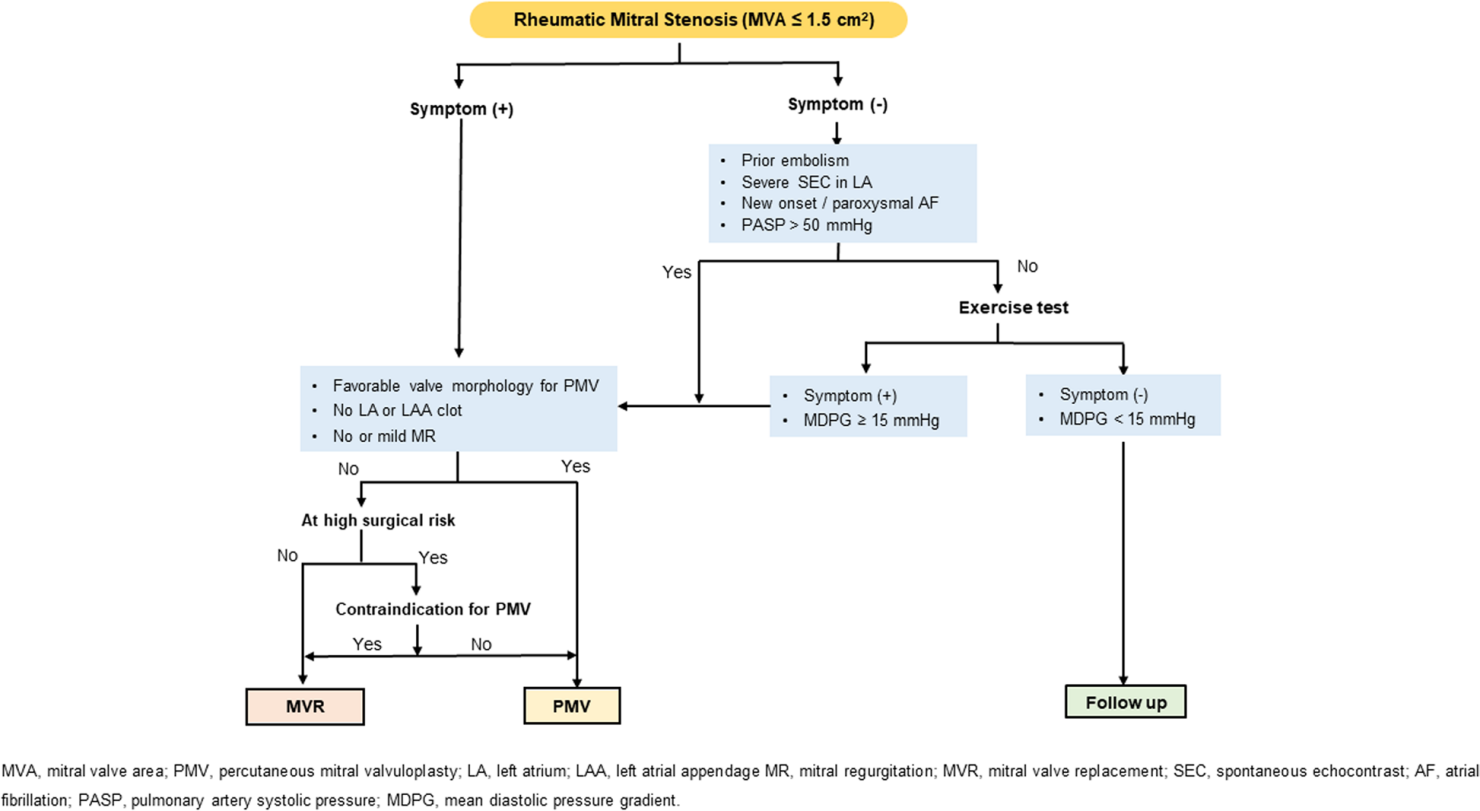
Treatment of rheumatic mitral stenosis. AF, atrial fibrillation; LA, left atrium; LAA, left atrial appendage; MDPG, mean diastolic pressure gradient; MR, mitral regurgitation; MVA, mitral valve area; MVR, mitral valve replacement; PASP, pulmonary artery systolic pressure; PMV, percutaneous mitral valvuloplasty; SEC, spontaneous echocontrast

Determining the optimal timing for surgical or percutaneous interventions in patients with MS is important to avoid the risks of unnecessary early intervention and irreversible pulmonary hypertension and/or right heart failure due to delayed intervention. Although the natural history data of MS have a retrospective nature, are prone to selection bias, and are unclear regarding severity of stenosis, most of the data consistently show poor prognosis without intervention in symptomatic MS patients. The 10-year survival rate of symptomatic patients is 34% to 61% and the 20-year survival rate is 14% to 21%, which is very poor [46, 47]. In particular, the 5-year survival rate is 44%, the 10-year survival rate is 32%, and the 15-year survival rate is 19% in patients who were recommended but refused intervention [48]. The most obvious timing of intervention is when symptoms are present in patients with severe MS (MVA ≤ 1.5 cm^2^) (Fig. 1).

The 20-year survival is excellent in asymptomatic patients with MS, but half of the patients experience sudden deterioration due to AF or systemic embolism [47–50]. Early PMV was expected to have a clinical benefit compared to conventional management in patients with asymptomatic severe MS, but randomized control trials did not show that early PMV reduces cardiovascular events, including PMV-related complications, cardiovascular mortality, cerebral infarction, and systemic thromboembolic events, when compared to conventional management [29, 31]. In asymptomatic MS patients with AF or a previous thromboembolic event, early PMV reduced the composite endpoint including cardiovascular mortality, cerebral infarction, and systemic embolic events compared to patients with conventional treatment only [2]. Intervention should be considered in asymptomatic MS patients with previous systemic embolism, severe spontaneous echo contrast in LA or new-onset or paroxysmal AF.

Pulmonary hypertension is common, with a prevalence of 15% to 81% in patients with severe MS [51–54]. Pulmonary hypertension in patients with MS develops through postcapillary and precapillary hypertension. In the early stages of MS, pulmonary hypertension is caused by increased LA pressure, but as the disease progresses, pulmonary hypertension is exacerbated by increased pulmonary vascular resistance. Pulmonary arterial hypertension in patients with MS has an adverse effect on long-term prognosis [55, 56]. When there is clinical evidence of pulmonary hypertension in MS patients, the prognosis is poor in patients treated only medically [1]. Patients with severe pulmonary hypertension or with high pulmonary vascular resistance prior to the intervention had a higher mortality rate and less improvement in pulmonary hypertension after the intervention [57, 58]. It is reasonable to perform intervention for MS when pulmonary hypertension develops.

In general, intervention is performed only in patients with severe MS, because the risk of intervention outweighs its benefit in patients with MVA > 1.5 cm^2^. However, patients with an MVA > 1.5 cm^2^ may have unexplained symptoms for other reasons. This is related to the potential limitations of MVA measured by 2D planimetry or pressure half-time. In this case, intervention can be considered if MDPG increases over 15 mmHg through exercise tests. Conversely, in cases of severe MS with ambiguous symptoms, intervention may be considered if symptoms develop through exercise testing.

Intervention timing for patients with degenerative MS is different from that for patients with rheumatic MS. Patients with degenerative MS have a high risk regardless of type of intervention because they have severe calcification, old age, and multiple comorbidities. Therefore, in patients with degenerative MS, intervention should be performed only in those with severe symptoms.

### Choice of intervention

Effective treatment in patients with rheumatic MS is PMV or surgery. The choice of intervention is determined based on the clinical condition of the patient, the anatomical features of the valve, and the experience of the institution (Fig. 1).

PMV should be considered primarily for intervention except in cases where PMV is contraindicated or inappropriate. Unfavorable clinical characteristics for PMV include old age, prior commissurotomy, severe symptoms (New York Heart Association class IV), permanent AF, and severe pulmonary hypertension [37]. Echocardiography is used to confirm the absence of anatomical contraindications for PMV and to predict outcomes after PMV. In order to perform PMV, the following conditions must not be met: thrombus in LA, moderate or more MR, severe or bilateral commissural calcification, no commissural fusion, severe aortic valve disease or severe tricuspid stenosis and regurgitation, or requirement of concomitant coronary artery bypass surgery. The anatomical features of MV evaluated by echocardiography are related to the outcomes after PMV. Most investigators use Wilkins score or Cormier score to predict outcome after PMV [37, 59, 60]. Patients with Wilkins score greater than 8 showed suboptimal results [37]. Patients with valve morphology corresponding to Cormier groups 2 and 3 were 2.2 and 5.3 times, respectively, more likely to have inadequate results than those with valve morphology corresponding to group 1 [59]. PMV is also recommended if the patient has contraindications to surgery or is at high risk. In patients who underwent a successful PMV procedure, both the Inoue technique and the double balloon technique showed good long-term prognosis, regardless of the type of procedure [61]. A pre-PMV echocardiographic score > 8 and an immediate post-PMV MVA ≤ 1.76 cm^2^ are independent prognostic predictors of poor long-term prognosis, including MV reintervention, stroke, and cardiovascular death during a follow-up period greater than 10 years [8]. In a recent multicenter registry comparing the outcome of PMV and MV replacement, the treatment method did not affect cardiovascular mortality or heart failure hospitalization, but in very severe MS, early redo-intervention was found to increase in the PMV group [62].

In patients with symptomatic severe MS, PMV is recommended if the valve morphology is favorable for PMV, there is no clot in the LA or left atrial appendage, and significant MR is absent. However, if all the requirements for PMV are not met, MV replacement should be considered if the surgical risk is not high. In cases of high surgical risk, PMV may be considered in patients with resolved contraindications. For example, if thrombus in the LA or left atrial appendage disappears after anticoagulation, PMV can be done if other conditions are suitable for PMV. In patients requiring MV replacement, a mechanical valve is usually implanted because the durability of the prosthesis is better in mitral lesions and most patients require lifelong anticoagulant treatment due to AF.

In degenerative MS, unlike rheumatic MS, commissural fusion or leaflet involvement is rare, and calcification of the mitral annulus expands to the base of the leaflet, causing stenosis. Therefore, PMV or surgical commissurotomy has no role in patients with degenerative MS.

## Mitral regurgitation

### Etiology

MR is the most common valvular heart disease and is associated with excess mortality [63, 64]. The etiology of MR is divided mainly into primary MR and secondary MR. Primary MR is caused by primary anatomical and structural problems of the valve leaflets, chordae tendineae, and papillary muscles. The etiology of chronic primary MR includes the following: (1) MV prolapse (MVP) due to myxomatous change or fibroelastic deficiency; (2) degenerative leaflet thickening with calcification; (3) rheumatic change; and (4) congenital abnormality. MVP can occur in association with connective tissue diseases such as Marfan syndrome or Ehlers-Danlos syndrome [65]. In addition, the pathophysiology of primary MR due to MVP in patients with hypertrophic cardiomyopathy is associated with intrinsic MV abnormality and increased sheer stress [17, 65]. Papillary muscle rupture following myocardial infarction and destruction of MV leaflets due to bacterial endocarditis are the major causes of acute primary MR.

Secondary MR occurs by LV pathology, such as LV systolic dysfunction and enlarged LV size, resulting in impaired coaptation of MV due to the papillary muscle and chordae tendineae pulling the MV and forming MV tenting without problems in the MV apparatus [66]. Secondary MR can be caused by any cause of LV systolic dysfunction. It can occur not only from ischemic cardiomyopathy, but also from nonischemic cardiomyopathy, stress cardiomyopathy, and myocarditis. Of these, secondary MR caused by ischemic insult to the myocardium is called ischemic MR. Secondary MR can be caused by LA pathology, without an LV cause. LA enlargement and dilated MV annulus also result in impaired coaptation of MV, which is called atrial functional MR and is frequently observed in AF and heart failure with preserved ejection fraction [3].

### Stages

The stages of MR are based on valve anatomy and valve hemodynamics, and the criteria for severe MR in primary MR and secondary MR are the same: vena contract width (VCW) ≥ 7 mm, effective regurgitant orifice area (EROA) ≥ 0.4 cm^2^, regurgitant volume ≥ 60 mL, and regurgitant fraction (RF) ≥ 50% [1]. Table 4 demonstrates the stages of chronic primary MR and Table 5 demonstrates the stages of secondary MR.

**Table 4:** Stages of chronic primary mitral regurgitation

Characteristic	Stage					
	A	B		C1	C2	D
Definition	At risk	Progressive		Asymptomatic severe		Symptomatic severe
Severity	Normal to insignificant	Mild	Moderate	Severe		
Echocardiography morphology						
Leaflet	Mild thickening or restriction	Moderate to severe thickening or restriction		Severe thickening or restriction		
Prolapse	Mild	Moderate to severe		Severe		
Coaptation	Normal	Moderate to severe loss		Severe loss		
VCW (mm)	< 3	3–7		≥ 7		
EROA (cm^2^)	< 0.2	0.2–0.3	0.3–0.4	≥ 0.4		
Regurgitant volume (mL)	< 30	30–44	45–59	≥ 60		
Regurgitant fraction (%)	< 30	20–39	40–49	≥ 50		
LVEF (%)	Normal	Normal		> 60	≤ 60	-
LVESD (mm)	Normal	Normal		< 40	≥ 40	-
LA size	Normal	Normal or ↑		↑		
LV size	Normal	Normal		↑		
PV flow	Systolic dominance	Normal or systolic blunting		Systolic flow reversal		
Symptom	None	None		None		DOE, EI

*VCW* Vena contracta width, *EROA* Effective regurgitant orifice area by 2D-PISA method, *Reg.* Regurgitant, *LVEF* Left ventricular ejection fraction, *LVESD* Left ventricular end-systolic dimension, *LA* Left atrium, *LV* Left ventricle, *PV* Pulmonary vein, *DOE* Dyspnea on exertion, *EI* Exercise intolerance

**Table 5:** Stages of secondary mitral regurgitation

Characteristic	Stage				
	A	B		C	D
Definition	At risk	Progressive		Asymptomatic severe	Symptomatic severe
Severity	Normal to insignificant	Mild	Moderate	Severe	
Echocardiography morphology					
Tethering	Normal to insignificant	Mild to moderate		Severe	
Coaptation	Normal	Limited		Severe loss	
Annulus	Normal	Dilation		Dilation	
VCW (mm)	< 3	3–7		≥ 7	
EROA (cm^2^)	-	0.2–0.3	0.3–0.4	≥ 0.4	
Regurgitant volume (mL)	-	30–44	45–59	≥ 60	
Regurgitant fraction (%)	-	20–39	40–49	≥ 50	
LA size	Normal or mildly ↑	↑		↑	
LV size	Normal or mildly ↑	↑		↑	
LV systolic function	Normal or ↓	↓		↓	
RWMA	Fixed or inducible	Presence		Presence	
PV flow	Systolic dominance	Normal or systolic blunting		Systolic flow reversal	
Symptom	None (or angina)	None (or angina)		None (or angina)	Angina, DOE, EI

*VCW* Vena contracta width, *EROA* Effective regurgitant orifice area by 2D-PISA method, *LA* Left atrium, *LV* Left ventricle, *RWMA* Regional wall motion abnormality, *PV* Pulmonary vein, *DOE* Dyspnea on exertion, *EI* Exercise intolerance

### Korean data

A recent nationwide hospital-based retrospective cohort study from the Korean Valve Survey collected clinical and echocardiographic data on valvular heart disease from 45 medical centers [28]. It showed that MR (1,384 of 4,089, 33.8%) is the most common significant valvular heart disease. Primary MR (*n* = 795, 57%) showed a higher proportion than secondary MR (*n* = 598, 43%); degenerative change (44.3%) and MVP (33.7%) were the most common causes of primary MR. In secondary MR, nonischemic etiology is observed in two-thirds (63.0%) and ischemic etiology in one-third (29.3%). The mean LV ejection fraction (LVEF) of secondary MR was 38.9%, and proportion of LVEF less than 40% was 57.2%.

A recent study showed distinct phenogroups of patients with primary MR undergoing valve surgery [67]. In this study, 1,629 patients who underwent valve surgery with severe primary MR were divided into five groups (group 1, least comorbidities; group 2, men with LV enlargement; group 3, predominantly women with rheumatic MR; group 4, low-risk older patients; and group 5, high-risk older patients) and the patient's prognosis was evaluated. The elderly and high-risk patients (group 5) had the poorest prognosis, while the young and low-risk patients (group 1) had the best prognosis [67]. It was suggested that defining the patient group would help to predict the patient's prognosis and determine appropriate surgical timing according to phenotype. Other studies evaluated prognosis by measuring LA strain and LV global longitudinal strain in severe MR patients who underwent MV surgery [68, 69]. Preoperative LV global longitudinal strain predicted the postoperative outcome and preoperative LA strain was also an independent predictor of long-term prognosis after surgery [68, 69]. After the Korean Ministry of Food and Drug Safety approved transcatheter edge-to-edge repair (TEER) use for degenerative MR in 2019, the initial experiences with TEER in Korea show that postimplantation MR grade ≤ 2 could be achieved in 94% and 30-day mortality rate was 6%, which were acceptable for initial procedural outcome [70].

Mortality and morbidity of secondary MR are high in heart failure patients with LV systolic dysfunction. Due to the lack of evidence for pharmacological therapy, the PRIME study was conducted to confirm whether sacubitril/valsartan improves secondary MR [71]. A total of 118 patients with secondary MR from four centers in Korea were enrolled in this study. The sacubitril/valsartan group had a more significant decrease in ERO and LV end-diastolic volume index than the valsartan group. The PRIME study showed that angiotensin receptor-neprilysin inhibitors may be considered for optimal medical therapy in stable patients with heart failure and functional MR.

### Diagnosis and follow-up

#### Primary MR

Evaluation of the pathologic change of MV apparatus (leaflets, chordae tendineae, papillary muscles, and annulus) and LV remodeling is crucial for grading and determining treatment strategy for primary MR. The presence of MR-related symptoms or arrhythmias such as AF should also be assessed to determine the optimal timing of MR intervention.

1. Diagnosis

TTE is a baseline imaging modality to diagnose and grade primary MR. Evaluation of pathoanatomic lesions of the MV causing MR is needed to identify a specific etiology and evaluate feasibility of surgical or transcatheter repair [72]. Parameters for evaluating MR severity include EROA, regurgitant volume, vena contracta, jet area by color Doppler, and transmitral jet velocity [73, 74]. Changes in LVEF, LV volume and pulmonary artery pressure should also be evaluated to guide the timing of MR intervention and predict long-term outcome. Although LVEF is a key factor in determining the timing of MR intervention, it is frequently load-dependent in patients with chronic MR. In this case, global longitudinal strain may be useful to reflect LV dysfunction more sensitively [75, 76]. When the MR is multijet or eccentric, 3D echocardiography may be helpful to accurately quantify regurgitant volume [77, 78]. In patients with primary MR, TEE is indicated for evaluation of severity and mechanism of MR when TTE provides inadequate image quality or discordant information. Especially, the *en face* view using 3D TEE can visualize the same inspection with a surgical view and therefore may be helpful for surgical planning. Cardiac magnetic resonance (CMR) provides a more accurate volumetric assessment of LV, LA, and LVEF than TTE or TEE. Thus, in cases where the echocardiographic image is too poor to measure chamber size, CMR may be indicated [75, 79, 80]. Although CMR is known as a gold standard for measuring regurgitant fraction in patients with MR, CMR is possible when the adequate quality of velocity-encoded imaging acquisition is ensured. Also, CMR is less useful for evaluating MV pathology. In asymptomatic patients with severe primary MR and nondilated cardiac chambers, patients who had low brain natriuretic peptide (BNP) levels showed more favorable outcomes than those with high BNP levels [81]. Therefore, checking BNP value can be useful for predicting the clinical course in asymptomatic patients with severe primary MR. Exercise echocardiography may identify the cardiac origin of dyspnea in patients with discordant symptoms and MR grade at rest [82, 83]. Changes in MR volume and pulmonary pressures during exercise is helpful in checking objective symptom and hemodynamic influence of MR [84, 85].

(2) Follow-up

The clinical situation (symptom onset, chest x-ray, electrocardiogram) and echocardiography should be assessed periodically in patients with chronic MR. In patients with asymptomatic MR, serial echocardiography is required depending on the MR severity. Reassessment of MR severity, LA/LV size, LVEF, and pulmonary artery pressure every 3 to 5 years in patients with mild MR, every 1 to 2 years in patients with moderate MR, and every 6 to 12 months in patients with severe MR are recommended. Recently, the Asian Valve Registry demonstrated that LV mass index was independently associated with development of MR-related symptoms [86]. Therefore, when the occurrence of cardiac symptoms due to MR is not convincing, change in LV mass index may help the decision-making. Measurement of BNP levels, exercise echocardiography, electrocardiogram and CMR may be considered complementary diagnostic and risk stratification tools [86–89].

(3) Medical therapy

In patients with acute MR, nitrates and diuretics are used to reduce filling pressures. Sodium nitroprusside reduces afterload and regurgitant fraction. However, there is no evidence to support the prophylactic use of vasodilators in chronic primary MR with preserved LVEF. In patients with overt heart failure, medical therapy is reasonable [90, 91].

(4) Timing of intervention

**Fig. 2:**
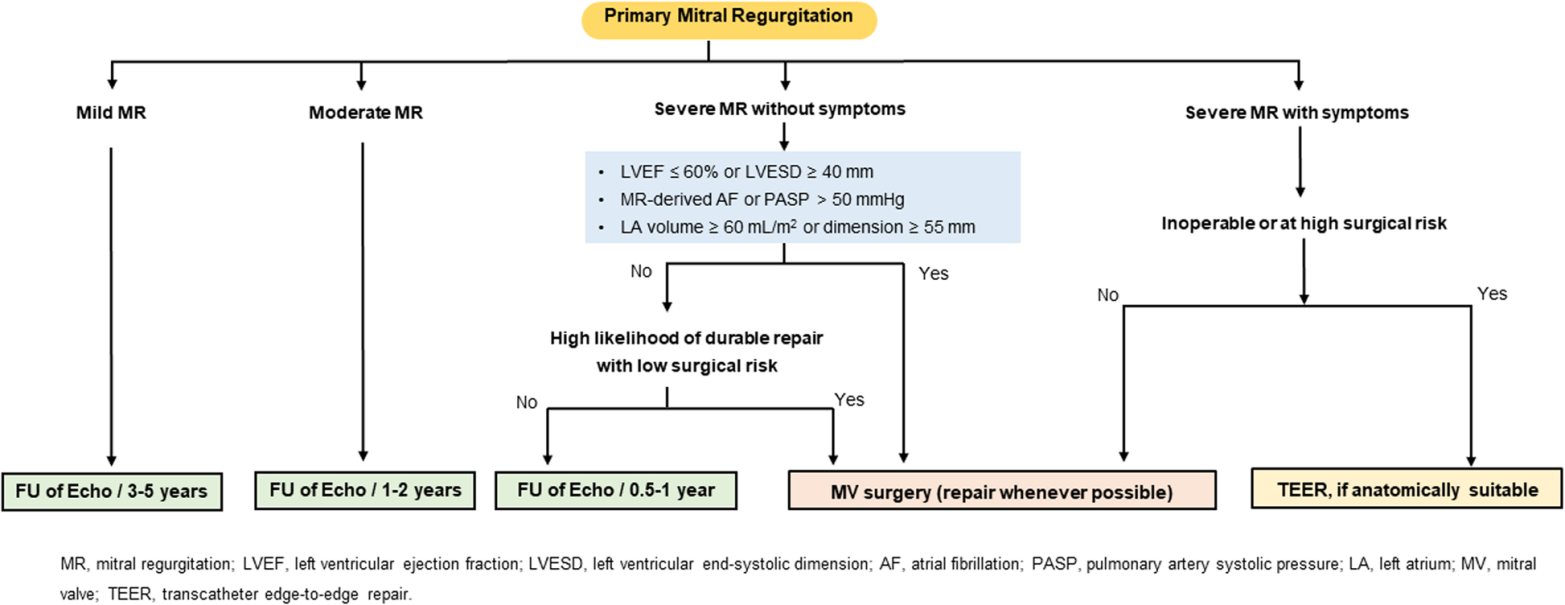
Treatment of primary mitral regurgitation (MR). AF, atrial fibrillation; FU, follow-up; LA, left atrium; LVEF, left ventricular ejection fraction; LVESD, left ventricular end-systolic dimension; MV, mitral valve; PASP, pulmonary artery systolic pressure; TEER, transcatheter edge-to-edge repair

Patients with acute severe MR, caused by acute chordae rupture, papillary muscle rupture, or infective endocarditis, usually present with decompensated symptoms and signs of heart failure, therefore, urgent surgical treatment is recommended in acute severe MR. Indications for intervention in chronic primary MR are shown in Fig. 2. In symptomatic patients with severe primary MR, MV surgery is recommended irrespective of LV systolic function. The benefit of early surgery in asymptomatic patients with severe primary MR has been suggested in prospective, observational studies [92–94]. Especially in asymptomatic patients with severe primary MR and preserved LVEF over 50 years of age, the efficacy of early surgery on reducing cardiac mortality was much more significant than conservative management [94]. In this regard, imaging surveillance is useful to determine the ideal time for MV surgery before the development of LV systolic dysfunction. The presence of LVEF ≤ 60%, LV end-systolic dimension ≥ 40 mm, LA volume index ≥ 60 mL/m^2^ or diameter ≥ 55 mm, systolic pulmonary arterial pressure > 50 mmHg, and AF are considered triggers for intervention regardless of symptomatic status [95–99].

(5) Choice of intervention: surgical repair and replacement

When the surgical treatment is determined, MV repair should be considered in preference to replacement under multidisciplinary discussion by a heart team. Large prospective observational data have shown that MV repair is associated with lower operative mortality, better long-term survival, and fewer valve-related complications compared with MV replacement [100–102]. Excellent surgical and cardiac outcome of mitral repair was observed regardless of the type of annuloplasty ring in the prospective randomized data in Korea [103]. However, in patients with advanced rheumatic MR, durability of MV repair can be limited by thickened or calcified leaflets, or extensive subvalvular disease [104]. Therefore, durability and success of repair versus replacement in rheumatic MR may be assessed by a comprehensive valve center.

(6) Choice of intervention: transcatheter MV repair

Studies of mitral TEER have demonstrated improvement of symptoms and a reduction in MR by 2 to 3 grades, leading to reverse remodeling of the LV in patients with symptomatic severe primary MR [105–107]. However, since surgical repair is the standard therapy in primary MR, TEER can only be considered in symptomatic patients with primary MR with high surgical risk and who are anatomically suitable for clipping. The 5-year follow-up data of TEER with MitraClip (Abbott) in patients with severe symptomatic primary MR are now available. Patients treated with percutaneous repair more commonly required surgery for residual MR during the first year after treatment [105]. However, between 1- and 5-year follow-ups, comparably low rates of surgery for MV dysfunction with either percutaneous or surgical therapy were reported [106, 107]. However, long-term follow-up data on the durability of TEER are needed. In addition, before determining TEER, a multidisciplinary discussion by the heart team regarding feasibility of MV anatomy for TEER and patient’s life expectancy should be considered.

#### Secondary MR

1. Diagnosis

Secondary MR is the condition of MR without significant pathologic problems of the leaflets [1, 2]. It results from LV dysfunction or remodeling by ischemic or nonischemic cardiomyopathies or atrial disease. Structurally, papillary muscular displacement with leaflet tethering and/or mitral annulus dilatation are the main pathologies [3, 108]. The most important diagnostic tool is TTE to identify the etiology and to assess severity of MR, LVEF, chamber sizes, and hemodynamic information including pulmonary arterial pressure [1, 2]. Coronary CT angiography or invasive coronary angiography is often required to evaluate the presence of coronary artery disease. Stress nuclear imaging, positron emission tomography (PET), CMR, or stress echocardiography can be considered to assess coronary artery disease as well as myocardial viability [109]. The utilization of TEE in secondary MR is focused on transcatheter valve interventions. It is indicated to determine suitability of transcatheter valve intervention or for guidance during the procedure [1, 2].

The definition of severe secondary MR is not different from primary MR. The recommended definition of severe secondary MR is EROA ≥ 0.4 cm^2^, regurgitant volume ≥ 60 mL, and regurgitant fraction ≥ 50% [1, 2]. Clinical evidence from previous studies indicates that secondary MR is associated with poorer outcomes compared to primary MR, even with smaller EROA [110, 111]. In addition, the discrepancy of estimated echocardiographic parameters may lead to difficulties in defining severe secondary MR due to following reasons: the total forward LV stroke volume is lower which can cause lower estimated regurgitant volume (< 60 mL); the crescent shape of the regurgitant orifice in secondary MR may lead to underestimation of the vena contracta width as well as EROA. Furthermore, multiple components other than the severity of MR can influence the prognosis of secondary MR, implying that the severity should not be determined only by the prognosis of the disease.

Multimodal imaging assessment is helpful in patients with secondary MR. Recent studies have demonstrated that CMR, global longitudinal strain, 3D echocardiography, and exercise echocardiography are additional useful tools to identify and prognosticate severe secondary MR [68, 79, 83, 112–116].

(2) Follow-up

The follow-up interval for TTE is recommended as follows based on the known MR progression rate and impact on the LV and LA. It is recommended to reassess MR severity, LA/LV size, LVEF, and pulmonary artery pressure every 3 to 5 years for patients with mild MR, every 1 to 2 years for patients with moderate MR, and every 6 to 12 months for patients with severe MR. Since secondary MR is often dynamic according to hemodynamic changes, it is recommended to perform a repeat TTE in the case of treatment that will change the size or function of the LV and LA. In addition to routine periodic imaging, the onset of symptoms or a change in the physical examination should raise concern about the cardiac response to the MR, necessitating a repeat TTE.

(3) Medical therapy

Chronic severe secondary MR with heart failure with reduced ejection fraction should receive four pillars of optimal guideline-directed medical therapy (GDMT), including angiotensin-converting enzyme inhibitors/angiotensin receptor blockers/angiotensin receptor-neprilysin inhibitors, β-blockers, aldosterone antagonists, and sodium glucose cotransporter-2 inhibitors, on top of adequate volume control with diuretics [90, 91]. The PRIME study showed that the reverse remodeling caused by angiotensin receptor-neprilysin inhibitors can reduce severity of secondary MR [71]. Other combined conditions that are related to heart failure need to be controlled according to the updated guidelines [90, 91]. Device therapy such as cardiac resynchronization therapy and optimal coronary artery revascularization are also helpful for reducing secondary MR.

(4) Timing of intervention

**Fig. 3:**
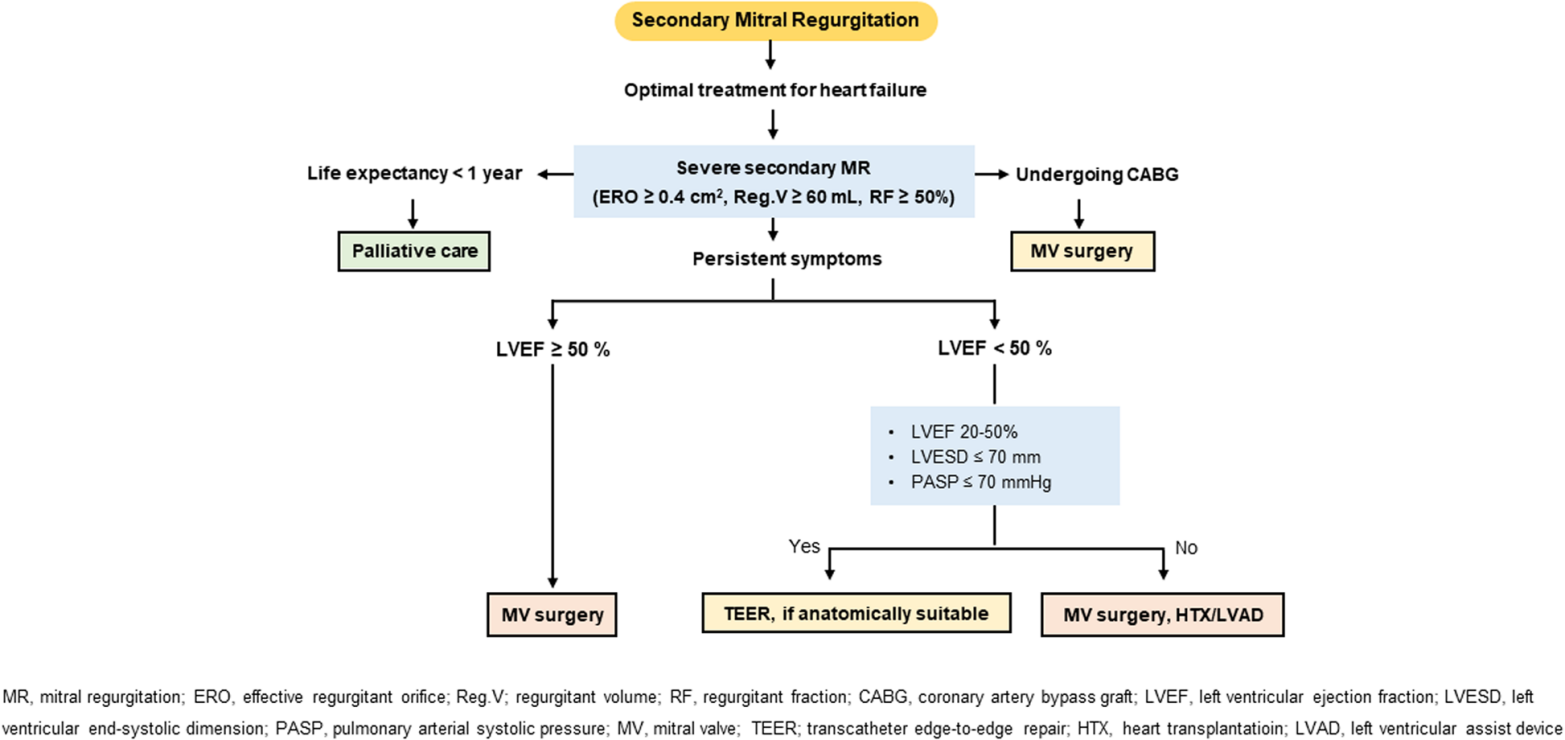
Treatment of secondary mitral regurgitation (MR). CABG, coronary artery bypass graft; ERO, effective regurgitant orifice; HTX, heart transplantation; LVAD, left ventricular assist device; LVEF, left ventricular ejection fraction; LVESD, left ventricular end-systolic dimension; MV, mitral valve; PASP, pulmonary arterial systolic pressure; Reg.V; regurgitant volume; RF, regurgitant fraction; TEER; transcatheter edge-to-edge repair

Figure 3 shows the decision-making process for secondary MR treatment. If symptoms persist after optimization of conventional heart failure therapy, further interventions should be evaluated before the deterioration of underlying LV disease [108, 117, 118]. The importance of decision-making by a multidisciplinary heart team consisting of a valve specialist, heart failure specialist, cardiac interventionist, and heart valve surgeon needs to be emphasized in this step [1, 2, 119]. When a patient has persistent symptoms despite the optimal treatment of heart failure, TEER or MV surgery is required according to the adequate indication [118, 120, 121]. For patients with severe comorbidities or life expectancy less than 1 year, palliative care could be considered.

(5) Choice of intervention

When patients with severe secondary MR and LV systolic dysfunction (LVEF < 50%) have persistent symptoms despite the optimal GDMT of heart failure, TEER with MitraClip system is reasonable provided that the appropriate anatomy is ensured (20% ≤ LVEF ≤ 50%, LV end-systolic dimension ≤ 70 mm, pulmonary artery systolic pressure ≤ 70 mmHg) [119, 121–124]. Two previous randomized controlled trials (COAPT and MITRA-FR) evaluated the safety and efficacy of TEER in patients with symptomatic heart failure and severe secondary MR. Both studies proved that the procedure is safe and effectively reduces severity of MR [120–123]. However, only the COAPT trial showed benefit in primary endpoint of all-cause mortality or heart failure hospitalization owing to the greater severity of secondary MR and lesser extent of LV dilatation (disproportionate severe MR) [118, 121–123].

The surgical approach needs to be personalized according to each circumstance. In patients with severe secondary MR who require coronary artery bypass graft due to myocardial ischemia, concomitant MV surgery is reasonable [93, 125–127]. In selected patients without advanced LV remodeling, MV repair with an undersized complete rigid ring can be performed to restore valve competence, improve symptoms, and reverse LV remodeling [102]. Chordal sparing valve replacement may be considered in patients with echocardiographic predictors of repair failure [126, 128, 129]. Isolated MV surgery in severe secondary MR needs special consideration owing to high combined risk, high recurrence rate, and the absence of proven survival benefit. In cases of atrial MR, which is often associated with AF, ring annuloplasty with AF ablation can be beneficial [3].

In severe secondary MR, optimal GDMT according to heart failure status should be ensured. When symptoms persist despite appropriate management, TEER or surgical MV repair/replacement can be considered. A multidisciplinary heart team approach is especially crucial in secondary MR due to the disease’s complexity.

## Tricuspid regurgitation

### Etiology

Less than moderate tricuspid regurgitation (TR) is frequent with normal tricuspid valve (TV) structure. However, significant TR is not uncommon. In a community-based study, moderate and severe TR was 5% and 0.6%, respectively [130]. In an asymptomatic Korean population on health checkup, moderate and severe TR was observed in approximately 0.3% [131]. TR was more prevalent in the elderly (75 years or more) and in women [131]. There is growing concern about TR with the increase in the aging population. Significant TR is also associated with poor long-term outcomes [132].

The etiology of TR can be divided into primary TR, caused by abnormal TV leaflets, and secondary TR, caused by tricuspid annular dilatation and leaflet tethering in the presence of normal leaflets (Table 6) [1]. Primary causes of TR involve the tricuspid apparatus. They include rheumatic disease, infective endocarditis, myxomatous disease, carcinoid syndrome, cancer involvement, radiation, drugs, congenital diseases such as Ebstein anomaly, chest trauma, and trauma including iatrogenic damage. Recently, significant TR due to cardiac implantable electronic device leads or endomyocardial biopsies is also increasing and can be found in 20% to 30% of the patients who underwent those procedures [133–135].

**Table 6:** Etiology of TR

Primary TR	Secondary TR
Rheumatic	Pulmonary hypertension with RV remodeling
Infective endocarditis	Primary pulmonary arterial hypertension
Myxomatous disease	Secondary pulmonary hypertension (left-sided heart disease)
Carcinoid syndrome	Tricuspid annular dilation (associated with AF)
Cancer involvement	RV volume overload (shunt/high output)
Radiation or drugs	RV dysfunction (cardiomyopathies or ischemic insult)
Congenital disease (e.g., Ebstein anomaly)	
Chest trauma	
Iatrogenic (CIED, endomyocardial biopsy)	

*TR* Tricuspid regurgitation, *RV* Right ventricle, *AF* Atrial fibrillation, *CIED* Cardiac implantable electronic devices

Secondary TR is more common (≥ 90%) and is mainly related to the setting of RV remodeling. RV remodeling is frequently induced by pressure overload due to pulmonary hypertension either primary or secondary to left-sided heart disease, volume overload due to left–right shunting, RV dysfunction related to cardiomyopathies or ischemic insult. Furthermore, around 10% of patients with secondary TR have “isolated” TR without significant pulmonary hypertension (pulmonary artery systolic pressure < 50 mmHg), with normal LV systolic function (LVEF > 60%) and no left-sided valves disease, and with normal appearing TV leaflets; “isolated” TR is mainly attributable to AF and dilatation of the right atrial (RA) and tricuspid annulus [1, 136]. Secondary TR accompanying RV remodeling is called “ventricular TR” and when remodeling of RA and annulus due to AF is dominant, it is called “atrial TR”. Secondary TR can develop late after left-sided valve surgery, mostly related to AF [137–139].

### Stages

The stages of TR are based on valve and annulus structure and valve hemodynamics, and the criteria for severe TR are VCW ≥ 7 mm, EROA ≥ 40 mm^2^, regurgitant volume ≥ 45 mL, and regurgitant fraction (RF) ≥ 50% [1]. Table 7 demonstrates the stages of TR. Severe TR is further classified into severe, massive, or torrential TR according to the degree of TR quantification [140, 141].

**Table 7:** Stages of TR

Characteristic	Stage								
	A	B		C			D		
Definition	At risk	Progressive		Asymptomatic severe			Symptomatic severe		
Severity	Normal to trivial	Mild	Moderate	Severe		Massive		Torrential	
Echocardiography morphology									
Leaflet	Normal	Mild to moderate thickening		Severe thickening, flail, perforation, retraction					
Coaptation	Normal, CIED, AF	Limited, prolapse, lead impingement		Severe loss, prolapse					
Annulus	Normal	Dilation		Dilation					
VCW (mm)	-	< 3	3–6	7–13		14–20		≥ 21	
EROA (cm^2^)	-	< 0.2	0.2–0.4	0.4–0.6		0.6–0.8		≥ 0.8	
PISA radius^a^ (mm)	-	≤ 5	6–8	≥ 9					
Regurgitant volume (mL)	-	< 30	30–45	45–59		60–74		≥ 75	
Regurgitant fraction (%)	-	< 30	30–49	≥ 50					
RA/RV size	-	Normal or ↑		↑					
Hepatic vein flow	-	Systolic dominance	Systolic blunting	Systolic flow reversal					
TR-CW Doppler jet	-	Faint/ parabolic	Dense/ parabolic	Dense, triangular					
Symptom	None	None		None		DOE, EI, right HF			

*TR* Tricuspid regurgitation, *CIED* Cardiac implantable electronic devices, *AF* Atrial fibrillation, *VCW* Vena contracta width, *EROA* Effective regurgitant orifice area by 2D-PISA method, *PISA* Proximal isovelocity surface area, *RA* Right atrium, *RV* Right ventricle, *CW* Continuous wave, *DOE* Dyspnea on exertion, *EI* Exercise intolerance, *HF* Right heart failure

^a^Radius at Nyquist limit shift of 28 cm/sec

### Korean data

A recent nationwide hospital-based retrospective cohort study from the Korean Valve Survey collected clinical and echocardiographic data on valvular heart disease from 45 medical centers [28]. Secondary TR accounted for 90% of TR in this valve survey. In Korean patients with moderate or more TR, half of the patients had AF or hypertension. Half of them had MR.

Korean researchers have conducted numerous studies on risk factors, imaging parameters, and prognosis of TR [142–154]. In a study of 299 patients who underwent successful PMV for MS, development of significant TR was associated with MV restenosis [142]. Age, female sex, rheumatic etiology, AF, and peak pressure gradient of TR at follow-up were the independent risk factors for the development of late significant TR after successful left-side valve surgery [138]. The degree of TR and some quantitative parameters of TR have been shown to predict adverse clinical outcomes. In a study of 429 patients who underwent TTE before and after pacemaker implantation, pacemaker-related TR was found in 9.8% and was associated with AF, history of open-heart surgery, and poorer cardiovascular outcomes [143]. In patients with severe isolated TR, VCW > 7 mm was a powerful independent predictor of adverse outcomes [144]. In another study that compared the VCW of 2D echocardiography with 3D echocardiography in functional TR, different VCW cutoff values were suggested because the cross-sectional shape of the VCW was ellipsoidal with a long anteroposterior direction [145]. In addition, it was demonstrated that VCW was determined by annular dilation and leaflet tenting in the corresponding directions. In a study with real-time 3D echocardiography, TR severity was determined by septal leaflet tethering, septal-lateral annular dilatation, and the severity of pulmonary hypertension [146].

In a total of 213 patients with moderate or severe TR, a cutoff value of 40 mm for tricuspid annulus was the best predictor for cardiovascular events [147]. In patients with severe isolated TR undergoing TV surgery, reduced RV global longitudinal strain [148], reduced peak atrial longitudinal strain [149], and preoperative RV free-wall longitudinal strain were associated with poor clinical outcomes [150]. In atrial functional TR, baseline RA enlargement, and RA area to RV end-systolic area ratio were strong risk factors for progression of atrial functional TR [151].

The role of CMR was also studied in TR. In 75 patients with severe functional TR, preoperative assessment of CMR-based RV ejection fraction provided independent and incremental prognostic information in patients undergoing corrective surgery for severe functional TR [152]. TR fraction by CMR was independently associated with adverse clinical outcomes in patients with heart failure with reduced ejection fraction [153].

Some imaging parameters were related to a better surgical approach. In 238 patients who underwent stand-alone TV surgery (repair, 132; replacement, 106) for severe TR, a trend favoring replacement was shown in patients with annular diameter > 44 mm compared to repair [154].

### Diagnosis and follow-up

#### Diagnosis

TTE is most important to determine the etiology and severity of TR as well as for routine follow-up [1, 2]. CMR imaging can be used as an auxiliary means for determination of the severity of TR, as well as for volumetric assessment and tissue characterization of the RV. Invasive cardiac catheterization for measurement of the pulmonary artery pressures, pulmonary vascular resistance, right-sided pressures, and cardiac index can be useful when clinical and noninvasive data are inadequate.

The severity of TR can be determined by a comprehensive consideration of multiple criteria, either semiquantitatively or quantitatively, as shown in Table 7. Mild TR usually does not have hemodynamic significance, while severe TR leads to RV and RA dilatation, remodeling, elevated systemic venous pressure, and eventually symptoms. While the current guidelines classify the severity of TR as mild, moderate, and severe [1, 2], there is increasingly a consensus that a spectrum of TR severity exists within the grade of “severe TR” which is inadequately reflected in the current grading scheme, especially with the advent of the transcatheter repair for TR. Thus, a new grading scheme increasing the grades to include massive and torrential TR has been proposed, using VCW and EROA [140, 141]. Due to the noncircular shape of the TR regurgitant orifice, measurement of the biplane VCW can be helpful [145]. The 3D regurgitant orifice area can be measured, but reported cutoffs have been diverging. The severity of TR can be dynamic and is affected by changes in preload and pulmonary artery pressure, and follow-up after optimal medical therapy should be done.

Besides the severity of TR, pulmonary artery pressures should be ascertained, either noninvasively with Doppler echocardiography or invasively with cardiac catheterization. Pulmonary artery systolic pressure can be estimated from the maximum TR velocity by continuous wave Doppler. However, the severity of pulmonary hypertension may be underestimated in patients with severe TR. In patients with suspected pulmonary hypertension of clinical significance or uncertain cause, invasive measurement of pulmonary artery pressures and pulmonary vascular resistance is essential for guiding treatment. The measurement of cardiac output using the thermodilution technique may be inaccurate with severe TR, and the Fick method using arterial and mixed venous oxygen content is recommended.

Also, annular dilatation is a risk factor for late TR in patients undergoing left-sided valve surgery. The tricuspid annulus should be measured in the RV-focused apical view at end-diastole, and it is reasonable for those with tricuspid annulus diameter > 40 mm (or 21 mm/m^2^) to receive concomitant TV repair at the time of left-sided valve surgery [1, 2]. RV function should be assessed, and normal RV systolic function is defined by several parameters including tricuspid annular plane systolic excursion > 16 mm, tricuspid annular systolic velocity > 10 cm/sec, and RV end-systolic area < 20 cm^2^ or fractional area change > 35% [1, 2]. RV strain and 3D echocardiographic measurements of the RV volumes can be considered to overcome the limitations of these conventional RV function indices [148, 150, 151]. CMR can provide more accurate volumetric measurements of the RV and assessment of RV function and tissue characterization [152, 155, 156].

#### Follow-up

The severity of TR and its consequences should be reassessed every 3 to 5 years in patients with mild TR, every 1 to 2 years in patients with moderate TR, and every 6 to 12 months in patients with severe TR. Similar to MR, TR is often dynamic according to volume status, changes in left-side chambers, and pulmonary artery systolic pressure. Therefore, it is recommended to perform a repeat TTE in case of significant changes in volumes and hemodynamic status.

### Medical therapy

Severe TR can lead to signs and symptoms of right-sided heart failure, such as peripheral edema, ascites, fatigue, and indigestion. Lifestyle changes such as a low-salt diet and support stockings can be helpful. Medical therapies for severe TR itself are limited, and the mainstay is diuretics [1, 2]. The appropriate use of diuretics is important to decrease volume overload in severe TR. Loop diuretics are integral in treatment, but use can be limited due to worsening low cardiac output in the presence of RV dysfunction. Adding aldosterone antagonists can be beneficial, especially as hepatic congestion from right-sided heart failure can promote activation of the renin–angiotensin–aldosterone system.

In cases of severe secondary TR, therapies to treat the primary cause can be useful and may mitigate TR [1, 2]. Pulmonary vasodilators for selected patients with pulmonary arterial hypertension can reduce pulmonary vascular resistance and resulting RV pressure overload. GDMT for patients with heart failure with reduced LVEF is effective for reducing RV volume and pressure overload and alleviating secondary TR. Rhythm control for AF may be helpful for preventing TR development related to tricuspid annular dilatation [139].

### Timing of intervention

**Fig. 4:**
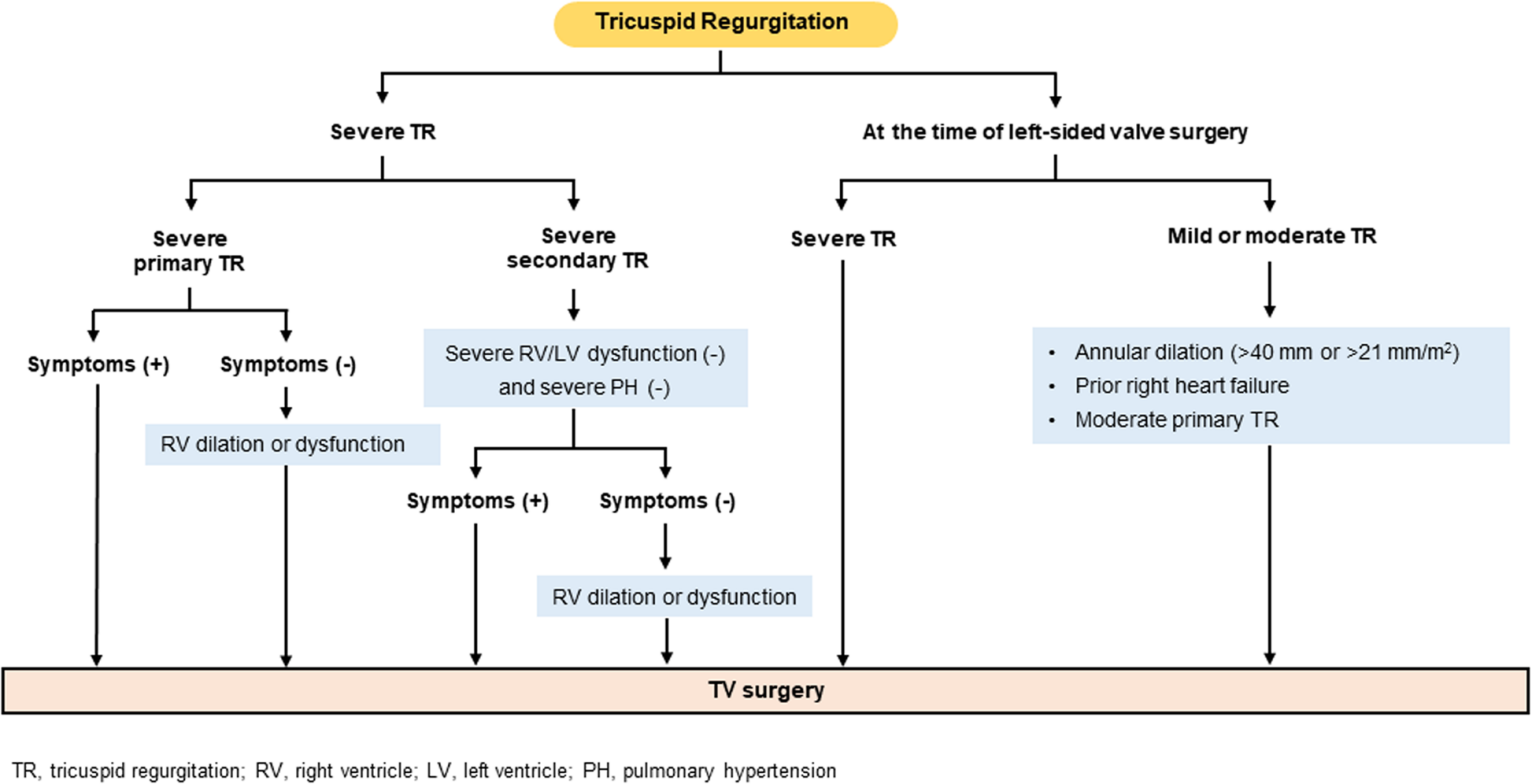
Treatment of tricuspid regurgitation (TR). LV, left ventricle; PH, pulmonary hypertension; RV, right ventricle; TV, tricuspid valve

Trivial or mild TR in patients with normal valves is commonly observed in routine echocardiography examinations and is of no clinical consequence. However, significant TR is associated with poor survival [157, 158]. Severe TR should be intervened before development of irreversible end-organ damage, such as advanced RV dysfunction, hepatic failure, and renal failure, which also increase surgical risk and affect postsurgical outcomes [159, 160]. Recommendations for intervention in TR are shown in Fig. 4. The latest ACC/AHA guideline [1] and ESC/EACTS guidelines [2] on the management of valvular heart disease mostly agree on the timing of intervention for TR, although the ESC/EACTS guidelines recommend earlier TV surgery for asymptomatic patients with severe TR [2].

In patients undergoing left-sided valve surgery, TV surgery should be performed liberally, especially considering the risk of late TR development [137–139]. In patients undergoing left-sided valve surgery, TV surgery is recommended in those with severe primary or secondary TR, regardless of symptoms [1, 2]. In these patients, TV surgery is also reasonable for those with mild or moderate TR and tricuspid annular dilatation (> 40 mm or 21 mm/m^2^), prior symptoms of right-sided heart failure, or moderate primary TR [1, 2].

In patients with symptomatic severe primary TR, TV surgery is recommended before the onset of severe RV dysfunction [1, 2]. In patients with asymptomatic severe primary TR with acceptable surgical risk, surgery should be considered when progressive RV dilatation or dysfunction is observed [1, 2]. However, exact thresholds have not been established. In otherwise healthy patients without other comorbidities and in the absence of RV dysfunction or pulmonary hypertension, the surgical risk associated with isolated TV surgery is low (< 1%–2% operative mortality) [1].

In patients with severe secondary TR, the benefit of isolated TV surgery is less well-established [161], and operative mortality is non-negligible, at around 10% [159, 162, 163]. However, with careful patient selection in the modern era, TV surgery can be performed with lower mortality than traditionally reported (< 4%–5%) [1, 164]. A prerequisite for consideration of TV surgery in severe secondary TR is the absence of severe RV and LV dysfunction and severe pulmonary hypertension [1, 2]: these patients should be treated medically due to the risk of postinterventional RV failure. Both the ACC/AHA guideline [1] and the ESC/EACTS guidelines [2] consider TV surgery to be reasonable in patients with symptomatic severe secondary TR. However, in patients with asymptomatic severe secondary TR, only the European guideline states that TV surgery is reasonable in those with RV dilatation [2]. In patients with previous left-sided valve surgery and symptomatic severe TR, isolated TV surgery is also reasonable and should not be delayed to improve the poor postoperative prognosis and high mortality rates related to reoperation and late referral [2].

### Choice of intervention

In TV surgery, valve repair, namely annuloplasty with prosthetic rings, is preferable to valve replacement whenever possible, and valve replacement should only be considered when there is severe TV leaflet tethering and annular dilatation or leaflet abnormality precluding valve repair [1, 2]. Observational data have shown that TV repair is associated with lower surgical risk than TV replacement, but there may be a selection bias, as TV replacement would be done in patients with a severely dilated annulus or abnormal leaflets [165, 166]. In the case of TV replacement, the selection of bioprosthetic or mechanical valve should be done considering the risk of lifelong anticoagulation and the risk of reoperation [167–169]. A recent prospective randomized trial of tricuspid TEER for severe TR showed that tricuspid TEER was safe for patients with severe TR, reduced the severity of TR, and was associated with an improvement of quality of life [170]. In Korea, clinical application of tricuspid TEER to high-risk patients in surgery is expected to begin in the near future.

## Conclusions

In the absence of guidelines tailored to the Korean population, clinical treatment has faced several limitations in dealing with MR, MS, and TR. This position paper on valvular heart disease complies research results in Korea and combines foreign guidelines to provide guidance on diagnosis and treatment of MS, MR, and TR. Based on this position paper, further interest and research on clinical unmet needs are warranted in the future.

## Data Availability

The datasets used and/or analyzed during the current study are included in this manuscript. Additional data used to support the findings of this review are also available in this published article.

## Abbreviations

ACC/AHA: American College of Cardiology/American Heart Association
AF: Atrial fibrillation
BNP: Brain natriuretic peptide
CMR: Cardiac magnetic resonance
CT: Computed tomography
D: Dimensional
ERO: Effective regurgitant orifice
EROA: Effective regurgitant orifice area
ESC/EACTS: European Society of Cardiology/European Association for Cardio-Thoracic Surgery
GDMT: Guideline-directed medical therapy
HIRA: Korean Health Insurance Review and Assessment Service
KSE: Korean Society of Echocardiography
LA: Left atrium
LV: Left ventricular
LVEF: Left ventricular ejection fraction
MAC: Mitral annular calcification
MDPG: Mean diastolic pressure gradient
MR: Mitral regurgitation
MS: Mitral stenosis
MV: Mitral valve
MVA: Mitral valve area
MVP: Mitral valve prolapse
PMV: Percutaneous mitral valvuloplasty
RA: Right atrial
RV: Right ventricular
TEE: Transesophageal echocardiography
TEER: Transcatheter edge-to-edge repair
TR: Tricuspid regurgitation
TTE: Transthoracic echocardiography
TV: Tricuspid valve
VCW: Vena contract width
